# Use of sedative drugs in specialist palliative care (iSedPall): a multi-modal intervention pilot study protocol

**DOI:** 10.1186/s40814-025-01627-3

**Published:** 2025-04-10

**Authors:** Christoph Ostgathe, Claudia Bausewein, Eva Schildmann, Jeremias Bazata, Maria Heckel, Saskia Kauzner, Carsten Klein, Sabine H. Krauss, Alexander Kremling, Manuela Schneider, Andreas Seifert, Kerstin Ziegler, Christian Jäger, Jan Schildmann

**Affiliations:** 1 Department of Palliative Medicine, CCC Erlangen – EMN, University Hospital Erlangen, Friedrich-Alexander-Universität Erlangen-Nürnberg (FAU), Erlangen, Germany; 2 Department of Palliative Medicine, LMU University Hospital, LMU Munich, Munich, Germany; 3 Institute for History and Ethics of Medicine, Interdisciplinary Center for Health Sciences, Martin Luther University Halle-Wittenberg, Halle (Saale), Germany; 4 Department of Criminal Law, Criminal Procedural Law, Commercial Criminal Law and Medical Criminal Law, Friedrich-Alexander-Universität Erlangen-Nürnberg (FAU), Erlangen, Germany; 5 Interdisciplinary Centre for Palliative Medicine, Medical Faculty, University of Augsburg, Augsburg, Germany; 6 Paderborn Centre for Educational Research and Teacher Education – PLAZ Professional School, Paderborn University, Paderborn, Germany

**Keywords:** Sedative drugs, Multi-modal intervention, Palliative care, Study protocol

## Abstract

**Background:**

The use of sedative drugs in specialist palliative care is common but presents challenges due to specific medical, ethical, and legal considerations. There is little to no assistance for administering adequate sedative drug doses, ensuring accurate documentation before and during sedation, or managing ethically and legally challenging situations. In 2021, the SedPall study group published recommendations on the use of sedative drugs in palliative care. The German Association for Palliative Medicine endorsed the dissemination of the recommendations nationwide. However, disseminating recommendations alone does not necessarily lead to changes in clinical practice. In the project “Development and piloting of a multi-modal intervention for the use of sedative drugs in specialist palliative care (iSedPall)”, we will develop a multi-modal intervention that implements these national recommendations into practical tools for healthcare professionals in specialist inpatient and home care settings. In the pilot study described below, we aim to test the feasibility of the multi-modal intervention, its appropriateness, and acceptability as primary feasibility outcomes of the multi-modal intervention. Additionally, we aim to assess the feasibility of measuring healthcare professionals´ confidence in using sedative drugs as an outcome indicator for a possible subsequent study.

**Methods and analysis:**

We will use a mixed-methods approach to develop and pilot a multi-modal intervention. The primary feasibility outcomes and formative evaluation of the implementation process will be explored using quantitative (retrospective cohort study, survey) and qualitative elements (focus groups, interviews). Additionally, we will pilot the measurement of healthcare professionals´ confidence in using sedative drugs as an outcome indicator through a pre-post survey. Four specialist palliative care services will pilot the complex intervention for nine months. Due to the complexity of the intervention, we will follow the principles of the MRC framework for complex interventions and will apply a Theory of Change approach. The intervention will include different elements to be used throughout the patients’ treatment in inpatient and home specialist palliative care considering medical, ethical, and legal aspects for the use of sedative drugs and intentional sedation. The evaluation of the overall feasibility and the decision about proceeding to an implementation study will be based on the integration of quantitative and qualitative data, according to our mixed-methods approach.

**Discussion:**

This project is the first attempt to translate national recommendations on best practices for sedative drug use into a multi-modal intervention and tests its feasibility. The study group identified potential risks and challenges related to the intervention´s feasibility, acceptability, and appropriateness in advance. To mitigate these risks, the study protocol is based on a theoretical framework, developed through a Theory of Change approach. Participatory elements and the involvement of different stakeholders are expected to enhance user acceptance and feasibility, potentially improving the development of supporting materials for sedative drug use in specialist palliative care while considering the interests of non-professionals.

**Trial registration:**

Registered in the German Clinical Trials Register, DRKS-ID: DRKS00027241; Registered: 10/12/2021; https://www.drks.de/drks_web/setLocale_EN.do.

**Supplementary Information:**

The online version contains supplementary material available at 10.1186/s40814-025-01627-3.

## Background

The use of sedative drugs and intentional sedation in specialist palliative care (SPC) [1] is frequent but challenging due to specific medical, ethical, and legal considerations, which vary depending on the setting and country [2, 3]. Given the lack of a standardized terminology and concept of “intentional sedation to relieve suffering in palliative care” [1]—commonly known as “palliative sedation”—it is often difficult in practice to clearly differentiate between the use of sedative drugs to relieve unbearable suffering by reducing a patient´s consciousness and treating refractory symptoms [4, 5]. In light of this, many healthcare professionals state an uncertainty in administering an adequate dose of sedative drugs proportional to symptom relief without shortening life [6, 7].

Appropriate and accurate documentation of all relevant decisions before sedation, as well as monitoring parameters during sedation, supports best practice use and ensures patient well-being [8, 9]. However, documentation requirements differ between institutions and templates are scarce in literature [10]. This goes along with inconsistent guidance on using sedative drugs in SPC and its monitoring [11–16]. Furthermore, data from the Palliative Sedation EU Horizon 2020 project show that there was no national guideline for Germany [17].

Possible ethically and legally challenging situations for instance relate to demands for intentional sedation by the patient himself, intentional sedation with indication “existential suffering”, or requests for assisted suicide [18, 19]. Since 2020, assisted suicide has no longer been punishable by law in Germany due to a decision of the Federal Constitutional Court, ruling 26 February 2020, file nr 2 BvR 2347/15, so clearly stating the intent behind using sedative drugs is of the utmost importance for a clear demarcation against other practices at the end of life [7]. As a result of the lacking assistance for those challenges, current (inter-)national data show considerable heterogeneity in clinical practice [20–25].

In 2021, the SedPall study group—comprising clinical (Palliative Medicine/Erlangen, Palliative Medicine/Munich), ethical (Medical Ethics/Halle), and legal (Medical Criminal Law/Erlangen) partners—published recommendations on the use of sedative drugs in German SPC [26]. The terminology within the recommendations distinguishes different types of sedative drug use in SPC and the recommendations support the identification of ethical and legal issues. The German Association for Palliative Medicine (Deutsche Gesellschaft für Palliativmedizin; DGP) endorsed the recommendations nationwide and also published an English version [27]. However, publishing recommendations alone does not automatically change clinical practice [28, 29].

The findings from the previous project (SedPall) highlighted potential contextual barriers that could hinder the implementation of the recommendations: varying staffing levels, absence of guidance on monitoring, caregiving responsibilities for family members in a home care environment, diverse documentation systems, and ethical, legal, and clinical complexities [1, 2, 30]. To improve clinical practice, it is crucial to operationalize the recommendations into practical tools for healthcare professionals working in specialist palliative inpatient and home care settings.

Therefore, the iSedPall study group—funded by the BMBF: 01GY2020A-C—will develop an intervention for promoting best practice in using sedative drugs and intentional sedation for SPC. The main aim of the following pilot study is to test the feasibility of the multi-modal intervention, its appropriateness, and acceptability as primary feasibility outcomes of the multi-modal intervention. Additionally, we aim to test the feasibility of the confidence of healthcare professionals in using sedative drugs as an outcome indicator for a possible subsequent study.

## Methods and analysis

### Design

We will develop and pilot a multi-modal intervention using a mixed-methods approach. The feasibility of the multi-modal intervention, its appropriateness, and acceptability as primary feasibility outcomes will be evaluated using quantitative (retrospective cohort study, survey) and qualitative elements (focus groups, interviews). As a potential outcome indicator for a subsequent study, the effect on palliative care professionals’ confidence in their practice using sedative drugs and intentional sedation will be measured using a pre-post design. We will adhere to the SPIRIT guideline on reporting interventional trial protocols [31] (see Additional file 1) and to the CONSORT extension for pilot and feasibility trials [32] (see Additional file 2).

### Setting

Two inpatient SPC units of tertiary healthcare and two specialist palliative home care teams (urban and rural areas) with a multi-professional team composition (physicians, nurses, other professions) constitute the pilot centers and will implement the multi-modal intervention as part of the pilot study.

### Patient and public involvement

Our participatory approach aligns with current knowledge on patient and public involvement in palliative care [33–35] and will incorporate the insights acquired during the previous research project (SedPall) [26]. While directly involving patients is not possible due to the severe disease situation, we will involve individuals (former informal caregivers of palliative patients, hospice volunteers, interested citizens) from the participatory research groups in Erlangen, Munich, and Halle. Individuals will engage in specific tasks within the local participatory research groups and will be encouraged to contribute their own topics, while participation and feedback will be closely monitored and evaluated.

Considering the development of the individual elements of the intervention specifically aimed at patients and/or their informal caregivers, we anticipate significant value in soliciting feedback and active participation from the participatory research group, particularly concerning these elements. For additional expertise, a scientific advisory board with (inter)national experts from the field representing different occupational backgrounds (law, ethics, medicine, nursing) will be established to ensure independent expert advice and external quality monitoring.

### Multi-modal intervention

Since the intervention is complex due to multiple interacting components, required stakeholder behaviours, various possible outcomes and flexibility in its use, we will follow the principles of the updated MRC framework for developing and evaluating complex interventions, referring to the phases “develop intervention” and “feasibility” [36–38]. Given the complexity, a Theory of Change approach will be applied to support the developmental process by anticipating the expected mechanism of change in the intervention’s interacting elements as well as important contextual factors that should be considered for the implementation [36, 39]. Details about the multi-modal intervention development process will be published elsewhere. As of now, the updated EAPC framework on palliative sedation is only available as preprint and will therefore not be considered for the intervention’s development [40].

The multi-modal intervention will include various elements to be used throughout patients’ treatment in both inpatient and home SPC considering medical, ethical, and legal aspects of sedative drug use and intentional sedation [1]. The elements of the intervention will be used separately or in combination, depending on the individual patient case and the evaluation of the professional healthcare team. The elements of the multi-modal intervention are described in detail in Table 1.

**Table 1:** Elements of the multi-modal intervention

Category	Elements	Aim
Medication	“Warning list” (decision support tool for evaluation of sedative effects)	To provide cut-off values for dose intervals and doses, which are expected to result in a continuous effect/defined depth of sedation for sedative drugs for a defined “standard patient”, based on the available evidence and expert consensus
	Recommendations regarding drug doses for initiating intentional sedation in SPC	To provide recommendations for selection of drugs and doses when starting intentional sedation in SPC, based on the available evidence and expert consensus
Information and consent	Information sheets for patients and legal representatives	To provide legally sound information for the use of intentional sedation in SPC practice and to support the decision-making process
	Checklist for physicians on information provision	To guide the conversation between physician and patient during information and the declaration of consent by the patients and/or legal representatives ensuring decisions are properly documented
	Handout for informal caregivers of sedated patients	To provide additional support for informal caregivers before or during the application of intentional sedation in SPC
Documentation	Documentation templates for health professionals in SPC and informal caregivers in home care settings	To guide documentation for different types of sedative drug use according to pre-existing documentation systems in various settings in SPC
Moral challenge analysis	Ethical screening tool	To guide the use of the moral challenges analysis tool
	Analyses of ethically challenging situations from the perspective of medical ethics	To support ethical reflection on the use of sedative drugs in different SPC settings, e.g., for ethical case discussions
	Checklists for deliberation on each analyzed ethically challenging situation	To support appropriate deliberation when dealing with ethically challenging situation
	Informational brochure for patients and informal caregivers	To prevent ethical challenges, reduce stress for informal caregivers and minimize misunderstandings
Supplementary material	Educational materials for healthcare professionals	To provide information on the terminology, the multi-modal intervention and its elements, as well as support for the use of the tools

*SPC* specialist palliative care

We describe the intervention and the implementation based on the TIDieR checklist [41] (see Additional file 3). The study protocol outlines the collection and analysis of qualitative and quantitative data in the context of the pilot study.

### Pilot study

#### Procedure

##### Phase 1: implementation and adaptation of the intervention

To ensure smooth implementation and mitigate possible barriers, we will support the implementation by the following activities for each pilot center:

- Kick-off meeting at the start of the implementation to introduce the elements of the intervention to healthcare professionals, organized by representatives of the study group at the pilot centers
- Self-training of healthcare professionals in using the elements of the intervention with educational material, organized by the pilot centers themselves during a period of 2 weeks
- Online-meeting to discuss questions of healthcare professionals about the elements of the intervention
- Regular meetings with lead physician and study nurse of the participating centers to get updates about the current status of implementation
- After approximately 1 month of using the intervention in clinical practice, we will organize an interdisciplinary retrospective case-based feedback session, online or face-to-face at each pilot center, drawing on 3–5 selected patient cases concerning sedative drug use. The researchers will use the cases to collect detailed feedback from physicians and nurses about possible barriers to usage and identify any requirements for general or site-specific adaptations of the multi-modal elements of the intervention.

The researchers will incorporate general and site-specific adaptations—if necessary—of the multi-modal intervention based on the feedback received from the pilot centers during Phase 1. These adaptations will then be documented and incorporated into the manual for future implementation of the intervention.

##### Phase 2: feasibility testing of the multi-modal intervention

Phase 2 will begin with a kick-off meeting at each pilot center to introduce the adaptations to the intervention. Similar to Phase 1, regular meetings will be scheduled, and on-site visits will be conducted to discuss patient cases for which the elements of the intervention were utilized and to ensure commitment to the intervention. The meetings will also serve to inform the formative evaluation of the implementation process, including any consequences for the multi-professional team and potential structural changes.

### Data collection

#### Primary feasibility outcomes and evaluation of process

Our main aim is to test the feasibility of the multi-modal intervention, its appropriateness, and acceptability as primary feasibility outcomes of the multi-modal intervention. The assessment methods are described in the following.

In Phase 1, the retrospective chart review will be pre-tested. All patients treated by the centers during the piloting will be included in the study and retrospectively assigned according to the patients’ medical charts to one of five groups regarding the application of sedative drugs: (1) no sedative drugs, (2) application of sedative drugs without sedating effects, (3) intentional sedation, (4) sedation considered, and (5) sedative effect as adverse effect of a drug. In the context of the retrospective cohort study, demographic data, the use of sedative drugs (e.g., name, number and dose of sedative drugs), the frequency of use of the individual elements of the intervention (extent of use), appropriate for the given type of use of sedative drugs (adherence to the intervention), and completeness of documentation will be assessed. These data will be continuously extracted by the pilot centers and provided to the study group regularly. 

In Phase 2, the actual intervention phase, a sequential mixed-methods design (see Table 2) will be applied incorporating quantitative (retrospective cohort study, survey) and qualitative methods (focus groups, qualitative interviews) [42]. The preparation and pre-testing of the instruments (see Table 2) will be conducted in collaboration with the participatory research groups and both external and internal health professionals. The survey data including measures of feasibility, acceptability, appropriateness according to Kien et al. [43, 44], and process evaluation [45], will be used to inform the development of interview guides for the focus groups. These data sources constitute the summative evaluation assessing feasibility as “the extent to which a new treatment, or an innovation, can be successfully used or carried out within a given agency or setting” [37], dimensions of acceptability defined as “the perception among implementation stakeholders that a given treatment, service, practice, or innovation is agreeable, palatable, or satisfactory” [37]—such as user acceptance, barriers to usage, and user experiences—and the appropriateness of the intervention in terms of its “perceived fit, relevance, or compatibility” for the specific practice setting [37]. Based on the Consolidated Framework for Implementation Research [45], we will evaluate and monitor the implementation process considering its impact on the multi-professional team and potential structural changes in the services through continuous exchange with the pilot centers (formative evaluation).

**Table 2:** Data collection

Method	Testing of	Participants/materials	Pilot phase
Online survey	Healthcare professionals´ confidence in using sedative drugs (pre-assessment)	Healthcare professionals of pilot centers (*n* = 50)	Phase 1
Retrospective chart review	Summative evaluation of feasibility: (1) frequency of use of individual intervention elements, appropriate to the given type of sedative drug use; (2) completeness of documentation	Piloting centers ’Patients´ medical charts: approximately *n* = 1.080 medical records	Continuously
Qualitative Interviews, semi-structured	Feasibility, acceptability, appropriateness	Informal caregivers, patients	Phase 2
Online-survey	1) Confidence of healthcare professionals in using sedative drugs (post-assessment); 2) Feasibility, acceptability, appropriateness; 3) Implementation process	Healthcare professionals of pilot centers (*n* = 50)	Phase 2
Focus groups	1) Perceived changes in confidence in their own practice using sedative drugs; 2) Feasibility, acceptability, appropriateness; 3) Implementation process	Center-specific: multi-professional health professionals of piloting centers (*n* = 4, with 5–10 participants each) Setting-specific: with physicians and nurses of piloting centers (*n* = 2, with 4–10 participants each)	Phase 2
*SPC* Specialist palliative care			

#### Decision on overall feasibility

Following our mixed-methods approach, we will integrate quantitative and qualitative data to evaluate the overall feasibility of the intervention and determine whether to proceed with an implementation study. Data of the quantitative measures of feasibility, acceptability, and appropriateness will serve as main criteria [44]. Based on literature regarding the evaluation of newly developed interventions, mean measure scores ≥ 3 will be interpreted as “good” outcome regarding feasibility, acceptability, and appropriateness [48, 49]. In addition, we will use the quantitative data from the retrospective chart review to understand the use of individual elements of the intervention and adherence to the intervention. The qualitative data (focus groups, interviews) will be used as source of evidence on overall feasibility and will be analysed in terms of general user experiences, facilitators and barriers to use, and potential unintended consequences.

#### Piloting of potential outcome indicator

Furthermore, we aim to test the feasibility of “the confidence of healthcare professionals in using sedative drugs” as outcome indicator for a possible subsequent study and therefore we will collect initial data for a future sample size calculation [46]. The selection of our piloted outcome indicator was based on interview results from the previous project [2] and defined by means of the Theory of Change process. Both a customized pre-post online survey based on the Health Professionals Competence Scales (HePCos) [50] and focus groups [51] will be used for data collection. After scoping the literature for possible instruments, we concluded that there is no rigorously developed survey tool adequate for our purpose.

See Table 2 for an overview of selected methods for evaluation of primary feasibility outcomes, implementation process and piloting of the potential outcome indicator.

#### Sample size justification

In line with relevant literature, we did not conduct a formal statistical power calculation [46, 47]. Based onclinical experience, we expect about 30 patients to be treated per month per pilot center, which should result in an estimated sample size of 1080 patients to be included in retrospective chart review in Phase 2. With a population of 100 staff members, we can estimate a 50% response rate to within 10.2% for the survey with 95% confidence. We estimate that four center-specific focus groups of 5 to 10 healthcare professionals each and two setting-specific focus groups of 4 to 10 healthcare professionals will be needed to reach data saturation.

### Data analysis

Researchers will process quantitative data in IBM SPSS Statistics 29 and will analyze them using descriptive statistics (e.g., means, medians, standard deviations, frequencies and percentages). Primary feasibility outcomes (acceptability, appropriateness, and feasibility), and the potential outcome indicator will be analyzed by calculating means of the respective measures. We will descriptively compare means of measures of feasibility, acceptability, and appropriateness between professions as well as of settings to give first indications for potential differences between these groups. Potential changes in confidence in using sedative drugs within professions over the course of the study will be assessed by descriptive comparisons as well. A statistician will support the data analysis.

Possible underlying factors of these findings will be further explored in the focus groups. We will analyze qualitative data using qualitative content analysis according to Schreier [52]. In the next step, quantitative and qualitative data will be integrated to inform the decision about the overall feasibility of the intervention. The findings will be presented to the pilot centers, members of our scientific advisory board as external experts, and members of the participatory research groups. A number of measures will ensure methodological rigor. We will perform training on interviews and qualitative data analysis for researchers. To ensure intersubjectivity, two researchers will collaborate in qualitative data analysis, employing a systematic and transparent coding approach, along with mutual interpretation of the findings.

We will apply the following measures for quality assurance of the focus groups in accordance with the COREQ checklist [53]: all consortium partners will collaborate in the development of topic guides. The selection of participants in the four pilot centers will be based on purposive sampling, to the extent feasible, and participant checking will be conducted. To maintain consistency across individual focus groups, topic guides will be utilized, interviewers will receive additional training, and the involved researchers will have regular discussions to review previous focus groups.

### Publication of findings

The German Association for Palliative Medicine (DGP) will endorse the publication of the multi-modal intervention. The consortium will publish the findings of the pilot feasibility study open access in scientific journals. Publications will adhere to the authorship eligibility guidelines of the German Research Foundation (DFG) [54].

## Discussion

To the best of our knowledge, this project is the first attempt to operationalize national recommendations on best practices for the use of sedative drugs into a multi-modal intervention addressing the gap between existing guidelines and their application in practice. Furthermore, the project aims to test the feasibility, acceptability, and appropriateness of the multi-modal intervention as primary feasibility outcomes, based on a Theory of Change [36, 39]. We expect a”ready-to-use” and feasible multi-modal intervention for best practice of the use of sedative drugs and intentional sedation in SPC, developed by an interdisciplinary research consortium with involvement of participatory research elements.

Currently, the intervention is limited to German-speaking countries, which may be a limiting factor. However, the recommendations on best practices in the use of sedative drugs are available in English, allowing for international feedback and comparison with other national strategies. The study group identified potential risks and challenges related to feasibility, acceptability, and appropriateness of the intervention in advance. Risks could be the intervention not fitting real-life settings, general challenges in implementing new routines in healthcare systems, and neglecting the perspectives and needs of users, in this case healthcare professionals, as well as those of patients and their families. To mitigate these risks, the study protocol is based on a theoretical framework, developed through a Theory of Change approach (to be published elsewhere), in which the expected mechanisms of change are anticipated and important contextual factors are considered.

The potential outcome indicator “confidence of healthcare professionals in using sedative drugs” which is piloted for the future measurement of the effectiveness of the intervention, was defined due to its practical relevance and after scoping the literature regarding validated instruments. To explore and understand the user context and develop practical tools based on the actual user needs, we do not want to check the sedation practice by assessing the adherence to best practice recommendations explicitly. By formatively evaluating the implementation process as well, we expect to gain insight into supporting and challenging factors independent of the intervention itself, such as necessary resources, institutional structures, and the climate of change, which will inform future implementation strategies of the intervention. The applied mixed-methods approach supports this by quantitative and qualitative data. The participatory elements and involvement of different stakeholders throughout the development and piloting process should support high user acceptance and feasibility of the intervention. This increases the likelihood of developing supporting materials for using sedative drugs and intentional sedation in palliative care while considering the interests of non-professionals.

## Supplementary Information

Additional file 1. Recommended items to address in a clinical trial protocol and related documents.

Additional file 2. Information to include when reporting a pilot or feasibility trial.

Additional file 3. Template for Intervention Description and Replication.

Additional file 4. Translation of ethics approval by the Ethics Committee of the Faculty of Medicine, FAU Erlangen.

Additional file 5. Translation of funding approval.

## Data Availability

The datasets that will be generated and analysed during the pilot study, will be not publicly available due to data protection reasons, but will be available from the corresponding author on reasonable request.

## Abbreviations

CONSORT: Consolidated Standards of Reporting Trials
COREQ checklist: Consolidated Criteria for Reporting Qualitative Studies
DGP: German Association for Palliative Medicine
iSedPall: Project acronym: intervention for the use of sedative drugs in specialist palliative care
MRC Framework: Medical Research Council
SedPall: Project acronym: From anxiolysis to deep continuous sedation - Development of recommendations for sedation in specialist palliative care (SPC) (BMBF, 01GY1702A-C)
SPC: Specialist palliative care
SPIRIT: Standard Protocol Items: Recommendations for Interventional Trials
TIDieR checklist: Template for Intervention Description and Replication
DFG: German Research Foundation

## References

[1] Kremling A, Bausewein C, Klein C, Schildmann E, Ostgathe C, Ziegler K. Intentional sedation as a means to ease suffering: a systematically constructed terminology for sedation in palliative care. J Palliat Med. 2022;25(5):793–6. 35073180 10.1089/jpm.2021.0428 PMC9081045

[2] Meesters S, Bazata J, Handtke V, Gehrmann J, Kurkowski S, Klein C. “It’s pretty much flying blind in the home care setting”: a qualitative study on the influence of home care specific circumstances on sedation in specialist palliative home care. Palliat Med Januar. 2023;37(1):140–8. 10.1177/02692163221128938 PMC9841818 36242514

[3] Morita T, Kawahara T, Stone P, Sykes N, Miccinesi G, Klein C. Intercountry and intracountry variations in opinions of palliative care specialist physicians in Germany, Italy, Japan and UK about continuous use of sedatives: an international cross-sectional survey. Bmj Open. 2022;12(4):14. 10.1136/bmjopen-2021-060489 PMC9036469 35459681

[4] Kremling A, Schildmann J. What do you mean by palliative sedation? BMC Palliat Care. 2020;19(1):147. 32967659 10.1186/s12904-020-00635-9 PMC7513316

[5] Schildmann E, Pörnbacher S, Kalies H, Bausewein C. „Palliative sedation“? A retrospective cohort study on the use and labelling of continuously administered sedatives on a palliative care unit. Palliat Med Juli. 2018;32(7):1189–97. 10.1177/0269216318764095 29557260

[6] Schildmann J, Schildmann E. Clinical and ethical challenges of palliative sedation therapy The need for clear guidance and professional competencies. Int J Clin Pract. 2013;67(11):1086–8. 24165423 10.1111/ijcp.12227

[7] Meesters S, Grüne B, Bausewein C, Schildmann E. “We don’t want to sedate him” - A qualitative interview study on intentions when administering sedative drugs at the end of life in nursing homes and hospitals. BMC Palliat Care. 2021;20(1). Verfügbar unter: https://www.scopus.com/inward/record.uri?eid=2-s2.0-85114839772&doi=10.1186%2fs12904-021-00832-0&partnerID=40&md5=e1394b60a0e12e9066c753f5acbdae25 10.1186/s12904-021-00832-0 PMC8439055 34517847

[8] Witkowski C, Kimmel L, Edwards E, Cosic F. Comparison of the quality of documentation between electronic and paper medical records in orthopaedic trauma patients. Aust Health Rev Publ Aust Hosp Assoc. A 2022;46(2):204–9. 10.1071/AH21112 34749881

[9] Wood DL. Documentation guidelines: evolution, future direction, and compliance. Am J Med. 2001;110(4):332–4. 11239858 10.1016/s0002-9343(00)00748-8

[10] Stiel S, Heckel M, Christensen B, Ostgathe C, Klein C. In-service documentation tools and statements on palliative sedation in Germany—do they meet the EAPC framework recommendations? A qualitative document analysis. Support Care Cancer. 2016;24(1):459–67. 26268785 10.1007/s00520-015-2889-0

[11] Schildmann EK, Schildmann J, Kiesewetter I. Medication and monitoring in palliative sedation therapy: a systematic review and quality assessment of published guidelines. J Pain Symptom Manage. April 2015;49(4):734–46. 25242022 10.1016/j.jpainsymman.2014.08.013

[12] Schildmann E, Schildmann J. Palliative sedation therapy: a systematic literature review and critical appraisal of available guidance on indication and decision making. J Palliat Med Mai. 2014;17:601–11. 10.1089/jpm.2013.0511 24809466

[13] Janssens R, van Delden JJ, Widdershoven GA. Palliative sedation: not just normal medical practice. Ethical reflections on the Royal Dutch Medical Association’s guideline on palliative sedation. J Med Ethics. 2012;38:664–8. 22811556 10.1136/medethics-2011-100353

[14] Abarshi E, Rietjens J, Caraceni A, Payne S, Deliens L, Van Den Block L. Towards a standardised approach for evaluating guidelines and guidance documents on palliative sedation: study protocol. BMC Palliat Care. 2014;13(1):34. 25028571 10.1186/1472-684X-13-34 PMC4099031

[15] Surges SM, Garralda E, Jaspers B, Brunsch H, Rijpstra M, Hasselaar J. Review of European Guidelines on Palliative Sedation: A Foundation for the Updating of the European Association for Palliative Care Framework. J Palliat Med. 2022;25(11):1721–31. 35849746 10.1089/jpm.2021.0646

[16] Rijpstra M, Vissers K, Centeno C, Menten J, Radbruch L, Mercadante S. Monitoring the clinical practice of palliative sedation (PALSED) in patients with advanced cancer: an international, multicentre, non-experimental prospective observational study protocol. BMC Palliat Care. 2023;22(1):8. 36709271 10.1186/s12904-022-01125-w PMC9883602

[17] Garralda E. Palliative sedation- an european perspective. Verfügbar unter: https://palliativesedation.eu/wp-content/uploads/2021/10/Palliative-Sedation_A-European-perspective-EGarralda.pdf#:~:text=sedation%20in%20palliative%20care:%20ESMO%20Clinical%20Practice%20Guidelines

[18] Bruce A, Boston P. Relieving existential suffering through palliative sedation: discussion of an uneasy practice: relieving existential suffering. J Adv Nurs. 2011;67(12):2732–40. 21627682 10.1111/j.1365-2648.2011.05711.x

[19] Schur S, Radbruch L, Masel EK, Weixler D, Watzke HH. Walking the line. Palliative sedation for existential distress: still a controversial issue? Wien Med Wochenschr 1946. 2015;165(23–24). Verfügbar unter:https://pubmed.ncbi.nlm.nih.gov/26628315/. zitiert 7. Oktober 2024 10.1007/s10354-015-0402-5 26628315

[20] Seymour J, Rietjens J, Bruinsma S, Deliens L, Sterckx S, Mortier F. Using continuous sedation until death for cancer patients: a qualitative interview study of physicians’ and nurses’ practice in three European countries. Palliat Med. 2015;29(1):48–59. 25062816 10.1177/0269216314543319 PMC4266692

[21] Klosa PR, Klein C, Heckel M, Bronnhuber AC, Ostgathe C, Stiel S. The EAPC framework on palliative sedation and clinical practice—a questionnaire-based survey in Germany. Support Care Cancer. 2014;22(10):2621–8. 24743852 10.1007/s00520-014-2192-5

[22] Abdul-Razzak A, Lemieux L, Snyman M, Perez G, Sinnarajah A. Description of continuous palliative sedation practices in a large health region and comparison with clinical practice guidelines. J Palliat Med. 2019;22(9):1052–64. 30939060 10.1089/jpm.2018.0372

[23] Maltoni M, Miccinesi G, Morino P, Scarpi E, Bulli F, Martini F. Prospective observational Italian study on palliative sedation in two hospice settings: differences in casemixes and clinical care. Support Care Cancer Off J Multinatl Assoc Support Care Cancer. 2012;20(11):2829–36. 10.1007/s00520-012-1407-x 22361826

[24] Schur S, Weixler D, Gabl C, Kreye G, Likar R, Masel EK, u. a. Sedation at the end of life - A nation-wide study in palliative care units in Austria. BMC Palliat Care. 2016;15(1). Verfügbar unter: https://www.scopus.com/inward/record.uri?eid=2-s2.0-84969256081&doi=10.1186%2fs12904-016-0121-8&partnerID=40&md5=6fab6fe323967b5a44fe2c59218f9c7f 10.1186/s12904-016-0121-8 PMC4868021 27180238

[25] Maréchal N, Six S, Clemmen E, Baillon C, Tack A, Bauwens S. Reporting of palliative sedation and use of opioids at the end of life in a Belgian University Hospital: a pilot study. J Palliat Med. 2022;25(5):742–8. 34756109 10.1089/jpm.2021.0113

[26] Ostgathe C, Bausewein C, Schildmann E, Bazata J, Handtke V, Heckel M. Expert-approved best practice recommendations on the use of sedative drugs and intentional sedation in specialist palliative care (SedPall). BMC Palliat Care. 2023;22(1):126. 37667303 10.1186/s12904-023-01243-z PMC10476406

[27] Forschungsverbund SedPall, Deutsche Gesellschaft für Palliativmedizin e.V. The use of sedative drugs in specialist palliative care. Berlin; 2023. Verfügbar unter: https://www.dgpalliativmedizin.de/images/20230328_Broschuere_SedPall_Eng_fin.pdf. zitiert 8. April 2023

[28] Prior M, Guerin M, Grimmer-Somers K. The effectiveness of clinical guideline implementation strategies - a synthesis of systematic review findings: The effectiveness of clinical guideline implementation strategies. J Eval Clin Pract. Oktober 2008;14(5):888–97. Verfügbar unter: https://onlinelibrary.wiley.com/doi/10.1111/j.1365-2753.2008.01014.x. zitiert 5. April 2022 10.1111/j.1365-2753.2008.01014.x 19018923

[29] Grimshaw J, Eccles M, Thomas R, MacLennan G, Ramsay C, Fraser C. Toward evidence-based quality improvement.: evidence (and its Limitations) of the Effectiveness of Guideline Dissemination and Implementation Strategies 1966–1998. J Gen Intern Med. 2006;21(2):14–20. 10.1111/j.1525-1497.2006.00357.x PMC2557130 16637955

[30] Journal onko. de. 2019. Sedierung bei unerträglichem Leiden in der letzten Lebensphase – Klinische und ethische Herausforderungen. Verfügbar unter: https://www.journalonko.de/artikel/lesen/sedierung_unertraeglichem_leiden_klinische_ethische_herausforderungen. zitiert 15. August 2023

[31] Chan AW, Tetzlaff JM, Gotzsche PC, Altman DG, Mann H, Berlin JA. SPIRIT 2013 explanation and elaboration: guidance for protocols of clinical trials. BMJ. 2013;346(jan08 15):e7586–e7586. 23303884 10.1136/bmj.e7586 PMC3541470

[32] Eldridge SM, Chan CL, Campbell MJ, Bond CM, Hopewell S, Thabane L. CONSORT 2010 statement: extension to randomised pilot and feasibility trials. BMJ. 2016;355:i5239. 27777223 10.1136/bmj.i5239 PMC5076380

[33] Johnson H, Ogden M, Brighton LJ, Etkind SN, Oluyase AO, Chukwusa E, u. a. Patient and public involvement in palliative care research: what works, and why? A qualitative evaluation. Palliat Med. 2020;35(1):0269216320956819. 10.1177/0269216320956819 PMC7797607 32912087

[34] Chambers E, Gardiner C, Thompson J, Seymour J. Patient and carer involvement in palliative care research: an integrative qualitative evidence synthesis review. Palliat Med 2019;33(8):969–84. Verfügbar unter: http://journals.sagepub.com/doi/10.1177/0269216319858247. zitiert 2. März 2023 10.1177/0269216319858247 PMC6691598 31250702

[35] D’Eer L, Quintiens B, Van den Block L, Dury S, Deliens L, Chambaere K, u. a. Civic engagement in serious illness, death, and loss: A systematic mixed-methods review. Palliat Med 2022 ;026921632210778. http://journals.sagepub.com/doi/10.1177/02692163221077850. zitiert 22. März 2022 10.1177/02692163221077850 PMC11910752 35287517

[36] Skivington K, Matthews L, Simpson SA, Craig P, Baird J, Blazeby JM. A new framework for developing and evaluating complex interventions: update of Medical Research Council guidance. BMJ. 2021;374:n2061. 34593508 10.1136/bmj.n2061 PMC8482308

[37] Proctor E, Silmere H, Raghavan R, Hovmand P, Aarons G, Bunger A. Outcomes for implementation research: conceptual distinctions, measurement challenges, and research agenda. Adm Policy Ment Health. 2011;38(2):65–76. 20957426 10.1007/s10488-010-0319-7 PMC3068522

[38] Craig P, Dieppe P, Macintyre S, Michie S, Nazareth I, Petticrew M. Developing and evaluating complex interventions: the new Medical Research Council guidance. BMJ. 2008;337:a1655. 18824488 10.1136/bmj.a1655 PMC2769032

[39] De Silva MJ, Breuer E, Lee L, Asher L, Chowdhary N, Lund C. Theory of change: a theory-driven approach to enhance the medical research council’s framework for complex interventions. Trials. 2014;15(1):267. 24996765 10.1186/1745-6215-15-267 PMC4227087

[40] Surges SM, Brunsch H, Jaspers B, Apostolidis K, Cardone A, Centeno C, u. a. Revised European Association for Palliative Care (EAPC) recommended framework on palliative sedation: An international Delphi study. Verfügbar unter: https://advance.sagepub.com/articles/preprint/Revised_European_Association_for_Palliative_Care_EAPC_recommended_framework_on_palliative_sedation_An_international_Delphi_study/22329289/1. zitiert 26. Juni 2023 10.1177/02692163231220225 PMC10865771 38297460

[41] Hoffmann TC, Glasziou PP, Boutron I, Milne R, Perera R, Moher D. Better reporting of interventions: template for intervention description and replication (TIDieR) checklist and guide. BMJ. 2014;348:g1687. 24609605 10.1136/bmj.g1687

[42] Creswell JM, Creswell JD. Research design: qualitative, quantitative, and mixed methods approaches. 5th ed. Los Angeles: SAGE Publications; 2017.

[43] Weiner BJ, Lewis CC, Stanick C, Powell BJ, Dorsey CN, Clary AS. Psychometric assessment of three newly developed implementation outcome measures. Implement Sci. 2017;12(1):108. 28851459 10.1186/s13012-017-0635-3 PMC5576104

[44] Kien C, Griebler U, Schultes MT, Thaler KJ, Stamm T. Psychometric testing of the German versions of three implementation outcome measures. Glob Implement Res Appl. S 2021;1(3):183–94.

[45] Damschroder LJ, Reardon CM, Widerquist MAO, Lowery J. The updated consolidated framework for implementation research based on user feedback. Implement Sci. 2022;17(1):75. 36309746 10.1186/s13012-022-01245-0 PMC9617234

[46] Lancaster GA, Dodd S, Williamson PR. Design and analysis of pilot studies: recommendations for good practice. J Eval Clin Pract Mai. 2004;10(2):307–12. 10.1111/j..2002.384.doc.x 15189396

[47] Whitehead AL, Sully BGO, Campbell MJ. Pilot and feasibility studies: is there a difference from each other and from a randomised controlled trial? Contemp Clin Trials. 2014;38(1):130–3. 10.1016/j.cct.2014.04.001 24735841

[48] Leyenaar JK, Arakelyan M, Acquilano SC, Gilbert TL, Craig JT, Lee CN. I-CARE: feasibility, acceptability, and appropriateness of a digital health intervention for youth experiencing mental health boarding. J Adolesc Health Off Publ Soc Adolesc Med. 2023;72(6):923–32. 10.1016/j.jadohealth.2023.01.015 36870901

[49] Ellis S, Geana M, Griebling T, McWilliams C, Gills J, Stratton K. Development, acceptability, appropriateness and appeal of a cancer clinical trials implementation intervention for rural- and minority-serving urology practices. Trials. 2019;20(1):578. 31590694 10.1186/s13063-019-3658-z PMC6781342

[50] Grebe, Christian, Schürmann, Mirko, Latteck, Änne-Dörte. Health Professionals Competence Scales (HePCoS)PCOS. Health Professionals Competence Scales (HePCoS) - Startseite. Verfügbar unter: https://www.hepcos.com/cms/. zitiert 13. Juli 2023

[51] Hudson P. Focus group interviews: a guide for palliative care researchers and clinicians. Int J Palliat Nurs. 2003;9(5):202–7. 10.12968/ijpn.2003.9.5.11490 12819597

[52] Schreier M. Qualitative content analysis in practice. Los Angeles: SAGE; 2012. 272 S.

[53] Tong A, Sainsbury P, Craig J. Consolidated criteria for reporting qualitative research (COREQ): a 32-item checklist for interviews and focus groups. Int J Qual Health Care. 2007;19(6):349–57. 17872937 10.1093/intqhc/mzm042

[54] Deutsche Forschungsgemeinschaft. Guidelines for Safeguarding Good Research Practice. Code of Conduct. 20. Verfügbar unter: https://zenodo.org/record/6472827 . zitiert 19. Juli 2023

