# Supplementary material for: Use of sedative drugs in specialist palliative care (iSedPall): a multi-modal intervention pilot study protocol
Source: Pilot Feasibility Stud. 2025 Apr 10;11:45. doi: 10.1186/s40814-025-01627-3 (PMC11984285; doi:10.1186/s40814-025-01627-3)
Supplement: Supplementary file 4 — Additional file 4. Translation of ethics approval by the Ethics Committee of the Faculty of Medicine, FAU Erlangen. [file 40814_2025_1627_MOESM4_ESM.pdf]

Proposal Nr 21_381 B 11/24/2021

Study Title: Development and piloting of a multimodal intervention for the recommended use of sedative drugs in specialist palliative care (iSedPall)

Applicant: Saskia Kauzner

Dear Ms Kauzner,

The Ethics Committee has considered your application of 09/27/2021 considering the submitted documents in attachment 1 and deliberated the proposal at the meeting on 10/12/2021. Additional information/revision was available at 10/04/2021.

The Ethics Committee raises no objections to the conduct of the study.

Translation: Maria Heckel, 10/05/2022

Dr. Maria Heckel, maria.heckel@uk-erlangen.de, 004991318542514
