# Supplementary material for: Use of sedative drugs in specialist palliative care (iSedPall): a multi-modal intervention pilot study protocol
Source: Pilot Feasibility Stud. 2025 Apr 10;11:45. doi: 10.1186/s40814-025-01627-3 (PMC11984285; doi:10.1186/s40814-025-01627-3)
Supplement: Supplementary file 5 — Additional file 5. Translation of funding approval. [file 40814_2025_1627_MOESM5_ESM.pdf]

Translation of marked passage of the funding approval:

Dear Sir or Madam,

1. amount of the grant/financing form and type/ earmarking/grant period/Payment schedule

On behalf of and with funds from the Federal Ministry of Education and Research (BMBF), we hereby award you, as the project executing agency, a further non-repayable grant of up to

€ 889,468.10 (grant without flat-rate project allowance) up to a maximum of the eligible expenses (full financing) plus a project fee of € 177,893.62 (20.00 % of the above-mentioned grant or of the BMBF-financed eligible expenses).

This means that the total grant, including the project allowance, amounts to 1.067.361,72 €

(in letters: One-zero-six-seven-three-six-one-comma-seven-two euros).

Translation: Maria Heckel, Erlangen, 05.10.2022
